# Periscope; or, Circumspect Review

**Published:** 1828-04-01

**Authors:** 


					1828] ( 433 )
OR,
CIRCUMSPECTIVE REVIEW.
[JANUARY 12, lg28.]
Under this division, we shall endeavour
to collect from the periodical press those
scattered fragments of practical or scien-
tific knowledge, which it would be as
difficult to set forth in separate articles
?f our Review, as it would be disadvan-
tageous to overlook. But how are we to
classify or arrange these multitudinous
contributions to our stock of information ?
The more we reflect on this subject, the
?iore the difficulties accumulate and be-
come insurmountable. Hie labor, hoc
opus. If ever there was a cycle or circle
in any science, it is in medical science.
Prom whatever point, in that circle, we
start, in practice, to that point must we
daily and hourly return?
Whatever link we strike,
Tenth, or ten thousandth, breaks the chain alike.
How is it possible to separate even the
study of morbid anatomy from that of
simple anatomy? Can we tell diseased
structure without comparing it with
healthy structure ? Can we separate the
treatment of a disease from an investi-
gation into its history, its etiology, its
symptomatology ? Or can we study any
of these advantageously, except in - con-
nexion with its pathology? Certainly
n?t. In the common routine of practice,
the whole circle of medical science comes
into play at every step we take?at al-
most every bed-side to which we are
called ! What physician is deserving of
that name, who is not intimately ac-
quainted with the effects of external
agents on the human frame, whether they
oe in the shape of moral impressions,
atmospheric influences, accidental vio-
lences, or surgical operations?a mental
misery, a malaria, a blow on the head, a
shattered limb, or an amputation ? What
surgeon is deserving of the appellation,
who is not as well acquainted with the
symptoms, causes, stages, diagnosis,
treatment, and morbid anatomy of perip-
neumony as of erysipelas ? The former
supervenes almost as frequently on the
flourishes of the catlin and saw, as does
the latter. As for the general prac-
titioner, if he had the hands of Briareus,
the eyes of Argus, and the ear of Diony-
sius, he would daily have occasion for
them all, in each and every branch of
medical science and practice.
Seeing, then, that these branches or
parts, to which we have given such learned
Greek titles, are locked and intersected
at every movement in our daily rounds,
how can we usefully classify them in this
our little microcosm ? On what plans
did Morgagni, Bonetus, Baillie, proceed
in their arrangements and classifications
of the "seats and causes," "the hidden
causes," (abditaecausae, Bonet.) the "mor-
bid anatomy," of diseases? On the plan
of the unlettered butcher in the sham-
bles ! The butcher first kills his patient,
viz. the sheep, and then strips off the
skin.* He next decapitates, and offers
the head separately to the public, then
the neck, the upper extremities, the con-
tents of the thorax and abdomen, the
pelvis, (saddle of mutton,) and lower ex-
tremities. So did Morgagni, Bonetus,
and Baillie. They could invent no other
arrangement or classification than that
of quartering and eviscerating the human
machine, presenting us with diseases of
the head, of the neck, of the thorax, the
abdomen, the pelvis, &c. as if all these
parts were independent and insulated
organs, whose derangements of function
and alterations of structure could be as
distinctly marked on the pathological
chart, as the 32 counties of England on
pasteboard ! What tyro does not know
the absurdity?the impossibility of these
unnatural insulations in actual practicc!
What man ever saw the brain "diseased,
* By this we do not mean to infer that
the doctor fleeces his patient before he
kills him. ^
Vol. VIII. No 16. 3 D
434 Periscope; or, Oiucumsbective Review. -[Jan. 12
without various other parts of the frame
being disordered ? Who ever sasv organic
disease of the heart, without disturbance
of function in the lungs ? We might
multiply the illustrations ad infinitum;
but enough has been said to justify us in
bringing together the disjecta membra
of this department, in the manner in
which they present themselves to us in the
book of nature, rather than in books of art.
We maintain, then, in the first place,
that the practice of medicine neces-
sarily includes a knowledge of surgery?
secondly, that the practice of surgery
necessarily includes the practice as well as
the knowledge of medicine?and thirdly,
that the practice of these two great
artificial divisions, necessarily includes a
knowledge of all the other divisions, how-
ever designated as subordinate, auxili-
ary, or collateral. Midwifery, pharmacy,
chemistry, medical botany, ophthalmic,
acoustic, and dental surgery, &c. are all
as arbitrary dislocations as those of the
unlettered butcher, the erudite Bonetus,
or the experienced Baillie. To these
separate branches let men attach them-
selves, (after being grounded by educa-
tion in all the others,) as interest, incli-
nation, or individual genius may prompt.
But, for our parts, we shall make no dis-
tinctions between the different members
of the same family?nor shall we impose
on ourselves any other rule than that of
selecting the grain from the chaff?and
thus separating useful facts from idle or
erroneous opinions.
Floriferis ut apes in saltibus omnia libant?
Omnia nos, itidein, depascimur aurea dicta.
PRACTICE OP MEDICINE AND
SURGERY.
In looking round the circle of the perio-
dical press, we are nearly as much em-
barrassed in the selection of a starting
point, as we were in respect to our no-
sological arrangement. Shall we begin
at home or abroad ? Shall we give the
post of honour to age, to size, to slow or
to rapid phases, to talent?to aristocracy,
or to democracy? Objections might be
urged against each of these modes of
setting out. In beginning at home, where
charity is said always to begin, we should
fail in politeness to our worthy con-
freres on both sides of the Atlantic?
and in giving a foreigner precedence, we
should offer a terrible violence to the pre-
judices of John Bull. In respect to
age, it is not every man?or woman either,
who likes to be put into the front rank
on that score. The size of a book is an
awkward claim to precedency. Indepen-
dent of the vulgar adage about the calf
and the veal, and the more classical adage,
" a great book is a great evil," there are
several other considerations that might
well deter us from placing the Goliahs
of periodical literature at the head of our
Periscope. In literary warfare, weight of
metal is of less importance than swift-
ness of sailing:?and water, as the source
of steam and vapour, is often more potent
than alcohol. As for talent, it is like
Hamlet's ghost?" hie et ubique." We
live in the very age of talent?" the march
of intellect." On every point of the me-
dical horizon, talent rises on our view.
We have journals of great talent?jour-
nals of little talent?journals of " all the
talent"?and (as anecessary consequence)
journals of?no talent at all. Talent,
therefore, is such a drug, that it will never
do as a qualification for precedency. It
only remains, then, to balance between
aristocracy and democracy. The for-
mer is too much encumbered with ancient
armour to lead the van?too much in-
flated with ancestral pride to bring up the
rear. As for the latter, decency forbids
that the sans culottes should be exposed
to the gaze of the world at the head of
our columns. In this dilemma about
precedency, we shall adopt the plan of
reverting to first principles?that of A,
B, C. In marshalling our conscripts on
the field, we shall summon them up in
strict alphabetical order, at a first in-
troduction, after which, we shall stand
on no ceremonies, in calling on them for
their respective contributions. The fol-
lowing journals are regularly received in
exchange, or purchased for the use of our
Periscope.
Annales de Med. Physiol. (Broussais.)
Archives Gdnerales
Bibliotheque Nouvelle.
Bulletin des Sciences Medicales.
Gazette (London Medical.)
Journal Complementaire.
Journal de Meddcine.
Journal de Physiologie. (Magendie.)
Journal de Progres.
Journal, (the Edinburgh Medical, Sec.)
1828]
M. Dupuytren on Traumatic Delirium. 435
Journal Universelle des Sciences Med.
Journal, (the Medical and Physical.)
Journal, (theNew YorkMed. andPhys.)
Literarische Annalen der Gesammten
Heilkunde.
Lancet.
Medico-Chirurgical Transactions.
Medical Recorder. (Philadelphia.)
Philadelphia Journal of the Medical
Sciences.
Repertoire G?n?rale.
Repository, (London Medical )
Revue Medicale.
Besides the above, we receive, though
not very regularly, two or three German,
and one or two Italian Journals. Let any
01>e glance his eye over this list, and say
whether or not a Periscope is necessary,
for the purpose of culling the more im-
portant information from these journals,
for English readers, not one in a thou-
sand of whom can get at the originals.
We have now only to remark, in con-
clusion, that it is our intention to review
the English periodicals within the phases
?f their publication, commencing with the
present year. A greater latitude must
be allowed for the foreign journals,
though Ave shall endeavour to bring up
?ur information to the latest moment.
In this review of journals, however, we
shall touch upon no matters but what are
useful, or injurious by being erroneous.
The former we shall condense, and trans-
fer, as far as is practicable, to our pages :
he latter we shall censure or disarm,
lr* as few words as possible. The great
mass of inert, harmless, or ponderous ma-
terials, with which our periodicals are
ballasted, rather than freighted, we shall
permit to float quietly down the stream
?ftime, to take their proper places, ac-
cording to their specific gravities, in the
?cean of oblivion.
On a Peculiar Species of Trauma-
tic Delirium.
Dupuytren and M. Helis, Hdtel Dieu.~\
The author justly observes, that it is not
Merely sufficient to prepare a patient for
a surgical operation, and to perform that
?peration adroitly ;?the more difficult
Part remains?the subsequent treatment.
-1 he moral and physical shock of an ope-
ration predisposes a persou peculiarly to
those accidents which naturally arise from
the wound, and the judgment of the prac-
titioner, on these occasions, is often of
more importance to the patient than his
manual dexterity while the knife was in
his hand. There are still some disciples
of Fiere Jacques in the surgical world.
"1 have cut you for the stone?may God
cure you." If trifling injuries and ope-
rations are sometimes followed by the
most serious constitutional disturbance,
how careful should we be, after great
operations, to guard the patient against
every source of supervening disease!
Sometimes we see deep-seated inflam-
mation arise in an organ or part to which
the knife has been applied?and the sym-
pathetic fever kindled up destroys the
patient, when success seemed certain.
At other times, a rigor announces the
formation of matter in some vital organ,
when the preceding fever was so masked,
or so trifling, as to throw the surgeon off
his guard. In other patients, the nervous
system becomes highly irritable after an
operation, and spasms or tetanus itself
ensue. In some, a species of delirium
succeeds the operation, which is of sin-
gular frequency and by no means devoid
of danger. Obscure in its causes, variable
in its march, but alarming in its symp-
toms, traumatic delirium is rarely fatal,
if treated judiciously and energetically.
The following cases are selected from a
great number of others, that occurred in
the wards of M. Dupuytren. The re-
flexions that are appended belong also
to the same illustrious surgeon.
Case 1. A young man from the coun-
try was operated on by M. Dupuytren,
in the month of June, for sarcocele, of
large size. He was much afraid of hae-
morrhage, and kept himself in a fidgetty
state all the day after the operation. On
the third day he was still more anxious,
and he was irritated by every motion,
gesture, or word of the neighbouring pa-
tients. Soon afterwards, he complained
of pain in his limbs and in his chest.
His eyes became animated?his breathing
hurried?he demanded food?and insisted
on getting out of bed. In short, he was
evidently delirious. His cries, the spai-k-
ling of his eyes, the immobility of the
pupils, the perspiration on his face, the
calm and regular pulse in the midst of
this eommotion, convinced M. Dupuytren
3D 2
436 Periscope; oit, Circumspective Review. [Ja?. 12
that the patient was affected with nervous
delirium (delire nerveux). Nevertheless
he examined the chest, of which he so
much complained, and found no disease
there. A few drops of laudanum were
immediately thrown up the rectum, and
the patient was secluded from all visitors,
so as to be kept quiet. In half an hour>
the patient fell into a profound sleep,
from which he did not awaken till the suc-
ceeding day, when he was completely tran-
quil. No accident afterwards occurred.
Case 2. An old man was operated on
for hernia by M. Dupuytren, and was
put to bed in the most promising state.
In a few hours afterwards, it was disco-
vered that he had torn open the wound,
in a fit of delirium, and actually lacerated
some portion of intestine, which he had
strained down ! He died in terrible tor-
ments, from the supervening peritonitis.
Case 3. A stone-mason fell from a
stage, and luxated the left femur. He
was taken to the Hotel Dieu, and M. Du-
puytren reduced the bone. The next
evening the patient was found in a state
of alarming agitation?his eyes glistening,
injected, and red?face flushed, and co-
vered with perspiration. He vociferated
aloud?tried to tear off the bandages?
and endeavoured to escape, as he said,
from the hands of justice. In the midst
of this disorder of the senses, the pulse
was regular, full, and of natural frequency
?the skin cool. The sister of the ward,
accustomed to these accidents, imme-
diately administered laudanum in injec-
tion, and tranquillity was quickly restored,
without any return of the nervous delirium.
Seven cases are detailed ; but, as they
all agree in the symptoms and treatment,
we need not pursue the relation. A long
train of reflexions, avowedly those of M.
Dupuytren, are appended. It is remarked,
that no surgical writer has taken notice
of this traumatic delirium, although it is
by no means uncommon. But one case
could be found in books. It was recorded
by M. Bouillon. The patient had been
operated on for popliteal aneurism, and,
on the fifth day, was seized with furious
delirium, sine febre, which lasted five
hours, and was removed by a laudanum
injection. The ligature, however, was
prematurely thrown oft', and the patient
died of hasmorrhage. Many surgeons,
indeed, have related cases, where patients
have torn open their wounds after ope-
rations, and thus destroyed themselves,
but none have investigated the cause of
this strange proceeding. Most surgeons,
too, have considered the phenomenon as
inflammatory, and, consequently, have
resorted to wrong measures. M. Dupuy-
tren thinks that, if left to itself, it would
only produce a temporary exhaustion;
but, in the mean time, irreparable injury
might be done to the wounds produced
by accident or operation. It is of great
importance to be able to foresee or pre-
dict the supervention of this curious ner-
vous commotion, and the author thinks
that, by the following signsr we may
prognosticate its approach.
If, then, within 12, 24, or 48 hours
after a fracture, a luxation, an attempt
at suicide, or a surgical operation, the
patient appears in unusually good spirits,
with talkativeness, glistening eye, and
quick movements?if he affect an unusual
degree of courage and resolution;?be on
your guard ! Do not irritate or excite
him?keep him quiet, and exclude all but
his nurses, as well as light and noise.
Very soon he will assail you with all
kinds of questions?he will flatter, me-
nace, abuse, sing, cry, and give way to
unceasing loquacity. This is not always
the most innocent kind of insanity. Our
author has known men in this state, get
up in the middle of the night, seize some
weapon, and commence a war of exter-
mination on all within their reach. He
has known others throw themselves out
of the windows, or commit suicide in
some horrible manner. The most re-
markable phenomenon, in these cases, is
the calmness of the circulation, and the
total absence of any febrile symptom. It
is a veritable mania, of short duration.
He has rarely known it last longer than
five or six days. The treatment is very
simple. Quietude, security, some lau-
danum, by way of injection. M. Dupuy-
tren prefers this mode of administering
the remedy greatly to that by the mouth.
He avers that, from six to ten drops, given
in this way, produce more effect than
triple the quantity, given by the mouth.
This ,-fact we have repeatedly witnessed
of late, and we could not have believed
it, had we not had the evidence of our
own senses in proof. It is probable, as
M. Dupuytren observes, that many medi-
1828] Fracture of Cervix Femoris. 437
cicines taken into the stomach are con-
siderably changed or neutralized by the
gastric juice and other matters with
which they must be necessarily mixed in
that digestive organ. In the lower bow-
els it is different?and the pure effects of
?pium may commonly be reckoned on
with more cei'tainty when applied to the
mtestinal surface at once. Here absorp-
tion, and not digestion, is the process
that is going forward. It is evident that
the former process is that which is most
favourable for the operation of medicines.
There is 110 doubt, too, that the impres-
sions made on the nerves of the lower
intestines are soon communicated to the
general nervous system. We shall not
advert to the speculations of Messrs.
I^upuytren and Sanson, 011 the nature and
proximate cause of this traumatic deli-
rium. The paper itself is very curious
and interesting.?Repertoire.
2. Fracture of Cervix Femoris.
[_Mr. Langstaff?Dr. Brulatour.]
Some considerable portion of the last
volume of the Medico-Chirurgical Trans-
actions is occupied with the almost worn-
out dispute about union of bone, in cases
of fracture of the cervix femoris within
the capsule. We have seen all the speci-
mens that have been presented at the
Society's meetings, and we freely declare
that there was not one which appeared to
us by any means unequivocal on the
affirmative side of the question. This was
also the opinion, as far as we could col-
let, of the majority of those members
present, who examined the specimens
and could make up their minds on the
subject. The specimens brought forward
hy Mr. Langstaff are, indeed, of the men-
dicity family?namely, beggars of the
question. Mr. L. had opportunities of
inspecting eight cases of the above acci-
dent after death, and he has " long been
satisfied of the possibility of union by
hone, from noticing the near approaches
Nature had made to effect this end in the
specimens he possesses." But why the
ossific union of fractures within the cap-
sule should be of such rare occurrence,
Mr. L. cannot divine, unless it be owing
to the difficulty of keeping the fractured
surfaces iu steady juxtaposition. The
case of Dr. James, an English physician,
resident at Bourdeaux, and transmitted
to the Society, together with the bone
itself, by Dr. Brulatour, was considered
by the ossific party as proof positive.
But unfortunately it wanted, as all the
others did, the sine qua non?the oppo-
site thigh-bone, in order to shew whether
the angles made by the cervix with the
shaft were different in the two sides.
Knowing the changes which naturally
take place in these angles by age, and
by inflammation, no dependence can be
placed on insulated specimens. The ac-
cident which happened to Dr. James had
doubtless produced considerable injury to
the coxo-fenioral articulation ; but the
appearances in the bone, and the want of
the other thigh-bone, for comparison,
rendered it impossible for any unpreju-
diced person to pronounce it a case of
ossific union after fracture within the
capsule. Our own opinion is, that this
was a case where the neck of the bone
was driven, as it were, into the head of
the shaft, by the fall, with consequent
shortening of the cervix. But this, we
opine, is a very different thing from a
fracture which completely separates the
articulating head of the bone from its
connexion with the rest of the femur.
P. S. When the above was written, we
received Mr. Bell's clinical lecture on
diseases of the hip-joint, as published in
the 4th number of the Med. Gazette,
where this eminent surgeon observes as
follows :?" When an injuiy has been
done to the hip-joint, and inflammation
follows, this process is apt to soften the
textures of the neck of the femur; and
consequently the head of the bone sinks
downwards, and is flattened, thus dimi-
nishing the length of the whole." He
then gives a wood-cut of this shortened
appearance, and difference in the angle
of the bone, exactly resembling that in
Dr. James's case, and several others that
have been produced before the Medico-
Chirurgical Society. Mr. Bell shews that
stiffness and lameness result from this
change, " because the head of the femur,
which naturally stands out free from the
trochanters, and thus permits extensive
and easy motion in the limb, is now by
the process of absorption of the cervix,
approximated to these processes, and
hence lameness results." We are glad
438 Periscope; or, Circumspective Review. [Jan. 12
to have the authority of Mr. Bell for the
strictures which we have deemed it our
duty to make on these supposed examples
of fracture with ossific union within the
capsule of the joint.
3. Moral and Pathological Effects
of Gambling.
In a late sitting of the Royal Academy of
Medicine, M. Gasc read a Memoir on the
above melancholy subject. This terrible
passion or propensity, is not so much out
of the boundary of the medical philoso-
pher's study as, at first sight, it may ap-
pear. Whatever raises a storm of con-
flicting passions in the human mind, must
induce a corresponding tumult in the or-
ganic functions, and thus lead to violent
disorders, fatal diseases, and not seldom
to self-destruction. M. Gasc conceives
that the propensity to gaming takes its
source in two of the most dominant pas-
sions of the human heart?self-love and
self-interest. Hence he accounts for
the habits of gambling in all ages and in
all nations, savage or civilized. Hence,
too, says he, the total inutility of the lec-
tures of the divine, the exhortations of
the--philosopher, and the penal statutes
of the legislator, in stemming the evil!
In depicting the effects of gaming on the
animal economy, M. Gasc exhibits the
gamester a prey, alternately, to delirious
joy, despair, and rage. It is no wonder
that the tremendous shocks which the
brain and nervous system must receive in
these paroxysms, should frequently des-
troy the intellectual faculties, and thus
?lead, as they actually do, to imbecility,
-insanity, and even furious mania. It is
?in the approaches to these conditions,
that the frequent acts of suicide are com-
mitted. The circulating system often
suffers in . these direful conflicts of the
passions, and aneurisms and other dis-
eases of the heart, are not seldom traced
to the gaming table. But no parts of the
animal economy suffer more directly and
unequivocally than the organs of diges-
tion?partly from the tortures of the
mind, which destroy appetite and sus-
pend digestion at once?and partly from
the stimulating potations which the
gamester swallows to support his courage
or drown his reflections !?Archives.
The foregoing facts have long been
familiar to the medical spectator; but
it is not so generally known to the pro-
fession?and it is but little known to the
public at large, how nearly the wide-
extended system of speculation, in this
country, approximates, in its ruinous
effects on the constitution, to gambling.
We lately saw a gentleman, of high pro-
bity, temperance, and respectability, who
mentioned to us the following fact, and
was curious to hear our explanation of it.
One day, on the Stock Exchange, when
the rumours of failures at home, and
commotions abroad, were producing such
terrible vacillations in the public funds
that his whole property was in momen-
tary jeopardy, he found himself in such a
state of nervous agitation, that he was
obliged to go out and apply to wine,
though quite unaccustomed to more than
a glass or two at his dinner. To his sur-
prise, the wine had no apparent effect,
and he drank glass after glass, in quick
succession, till a whole bottle was con-
sumed. Not the slightest inebriating
influence was induced by this unusual
quantity taken before his dinner. His
nervous agitation, however, was calmed,
and he went back to the Exchange, and
transacted his business with great stea-
diness and equanimity. None of the
common effects of wine were produced
at the time?but, the ultimate conse-
quence, several days afterwards, was a
severe attack of indigestion, to which he
had not previously been subject. Now,
this curious fact shews, we think, that,
although the mental agitation masks, or
even prevents, the common exhilarating
effects of wine and other stimulants at
the time, and thus induces and, indeed,
enables men to take more than under or-
dinary circumstances, yet that the ulte-
rior effects are worse on the constitution,
than if the stimulants had produced their
usual excitement at the moment of their
reception into the stomach. It is thus
that the nervous systems and digestive
organs of thousands in this country are
ruined, and that without the victims
being conscious of the channel through
which they are poisoned.
4. Injury of the Hip-joint.
[Mr. Stanley.?Med. Chir. Trans.~\
Mr. Stanley observes, that fracture of the
1828] M. Pelletieu on Traumatic Tetamus. 439
trochanter major, combined with fracture
of the cervix femoris, often bears a strong
resemblance to dislocation of the caput
femoris.
" Whenever the fractured portions of
the trochanter can be brought into con-
tact, a crepitus will be produced which
may enable the surgeon to ascertain the
precise nature of the injury. But when,
?from the direction of the fracture, one
portion of the trochanter has been drawn
by the action of the muscles towards the
great ischiatic notch, no crepitus may
then be discoverable, a direct source of
mistake will then arise from the positive
resemblance of the fractured portion of
the trochanter to the head of the femur,
the former occupying the same place
which the latter would do in dislocation;
and if, with these circumstances, there
should happen to be an inversion of the
injured limb, the difficulty of the diagnosis
must be considerably increased. This ob-
scurity, while it affords a strong motive
for extreme caution in such cases in our
own practice, should at the same time teach
us to be slow in citing a mistake in the
practice of others, as proving either igno-
rance or inattention
Mr. Stanley is nearly five years behind
the level of professional feeling on this
point. The fashion now is, to condemn
the practice of your contemporaries, whe-
ther it is good or bad?and the more
skilful they'are, the more copiously you
must pour on them the epithets of dolts,
ninny-hammers, &c. This is the way to
get on in the world. Mr. Stanley's ethics
will never do for the 19th century! But
?to return to injuries of another kind.
Case. A woman, aged 60 years, fell
on her right hip in the street. The limb
Was found slightly everted and shortened
three quarters of an inch ; yet it was
moveable in all directions. The extre-
mity of the shaft of the bone was in its
?natural situation, but behind the femur,
and at a little distance from it, a bony
prominence was discovered resting upon
?the ilium, towards the great ischiatic
notch, strongly resembling the head of
? the femur. Various opinions were enter-
tained as to the nature of the accident?
some considering it a dislocation, others
a fracture. After a confinement of some
months to her bed, the woman recovered
sufficiently to walk with the assistance of
a crutch, and in this state continued till
her death, three years afterwards. On
dissection, Mr. Stanley found that there
had been a fracture extending obliquely
through the trochanter major, and through
the basis of the neck into the shaft of the
bone. The prominence mistaken for the
head of the bone, was occasioned by the
posterior and larger portion of the tro-
chanter drawn backwards towards the
ischiatie notch.
Several other cases are related from
Mr. Stanley's practice, and some from
that of others. He concludes by observ-
ing that, in many cases where the nature
of the injury is doubtful, we ought to
impose on the patient a strict confine-
ment to his bed, for as great a length of
time as if the fracture had been ascer-
tained. The circumstances, attendant on
the accident should also be carefully in-
vestigated, as these are often our only/
guides.
5. On the Nature and Treatment or
Traumatic Tetanus.
[By M. Le Pelletier, Chief Surgeon of
the Hospital of Mans.]
The author of this Memoir appears to have
been placed in circumstances favourable
for the observation of facts and the inves-
tigation of phenomena?and he thinks he
can offer something more positive, as to
the nature and treatment of tetanus, than
has hitherto been given to the public.
M. Pelletier takes a course opposite to
those who consider tetanus as an affection
of the nervous system, and consequent-
ly as affording no appreciable physical
lesions as the cause of the symptoms.
He looks to the material and palpabte
alterations in the organs of the body, as
the basis of our investigations. He asserts
that, in all the dissections that have been
made of well-marked traumatic tetanus
in the Hospital of Mans, there were un-
equivocal traces of inflammation in the
vertebral meninges, especially at the ori-
gins of the nerves, and during their course
in the spinal column. Two cases are
narrated as a preliminary step, the bear-
ings of which will be readily seen.
Case 1. Nourri, aged 17 years, was
seized, on the 16th October, with head-
ache, fever, and pains in his joints, espe-
cially the hip-joints. After being a fort-
440 Periscope.; or, Circumspective Review. [Jan. 12
night ill in this way, without any medical
treatment, erysipelas came on the lower
extremities. A physician was called in,
and ordered leeches and blisters to the
inflamed parts?and cinchona internally.
By these means the patient's disorder was
increased, and he was sent to the hospital
on the 2d December. He was now in a
desperate condition, with symptoms of
engorgement in the head?chronic peri-
tonitis?and a kind of engorgement and
induration of the cellular tissue of the
lower extremities. We need not detail
the treatment. The symptoms were ame-
liorated, but, as the patient passed his
urine and stools in bed, he became ex-
coriated about the sacral region, ending
in ulceration which ultimately destroyed
life. But the most curious part of the
history is the supervention of trismus,
which made progress with the sacro-coc-
cygeal ulceration, till complete opistho-
tonos was established. It was concluded,
and perhaps with reason, that the disease
was traumatic tetanus, from the extension
of inflammation along the sacral nerves
to the coverings of the spinal marrow.
Our author had found copious general
bleeding the most effectual treatment in
other cases of traumatic tetanus ; but
here the patient was almost worn out
with preceding diseases. Nevertheless,
he ventured on venesection, which miti-
gated the symptoms, but did not arrest
the fatal course of the tetanus.
Dissection, The sacrum was denuded
in several points, as were the sacral nerves,
especially the sciatic, which shewed seve-
ral red spots externally, and red stri?
when slit up longitudinally. The neuri-
lema of the nerves was evidentlyinflamed.
The spinal marrow was laid bare (its
coverings uncut) throughout the whole
length of the vertebral canal. It pre-
sented externally several bluish streaks,
and its membranes appeared distended
by a fluid. There was, in fact, sero-san-
guineous infiltration beneath the mem-
branes?the pia matral covering was high-
ly vascular, so as to be as red as carmine,
particularly about the origins of the
nerves. The spinal marrow was not sof-
tened, nor did it present any mark qi
disease. The pia mater in the head was
rather injected, and there was some water
in the ventricles, but not of any magni-
tude,
Case 2. Pazon, aged 33 years, was
much bruised by the falling in of a quan-
tity of wood upon him, on the 18th May.
He was immediately carried to the hos-
pital, and the bone of one humerus was
found bi'oken in two or three places, with
a flesh wound above the elbow, produced
by the broken bone, which there pro-
truded. There was great pain and irri-
tation at this part. High fever and in-
flammation followed, and the usual an-
tiphlogistic measures were employed.
Suppuration and partial gangrene took
place, and, on the 26th, tetanus super-
vened. It is remarkable, if it be strictly
true, that the tetanic contraction of the
muscles commenced in the lacerated and
fractured arm?extended thence to the
pectoral muscles?to the muscles of the
jaw?and quickly destroyed life.
Dissection. The nerves of the brachial
plexus were carefully examined. The
neurilema of the cubital and median vein
was red and inflamed, and this inflam-
mation was unequivocal in the envelopes
of the spinal marrow and brain. It was
remarkable that the inflammation was
entirely confined to the left side of these
envelopes, namely, to the side corres-
ponding with the fx*actured arm.
Without indulging in extracts from our
author's reasonings, we may observe that
he comes to the conclusion, from the
above and many other dissections, that
tetanus is the result of inflammation?and
that the inflammation has its seat in the
neurilema of the nerves, and of the spinal
marrow?particularly in the pia matral
covering of this last organ.
From the above it will not be difficult
to come to the therapeutical indications
of M. Pelletier. If inflammation be the
pathological character of tetanus, it fol-
lows, of course, that depletion, in its
widest sense, must be the rational mode
of treatment. Unfortunately, the deple-
tive plan has too generally failed?but
this failure does not dispx-ove the patho-
logy of the disease maintained by our
author, and by several others, especially
Drs. Sanders and Reid, in this country.
It is wise to accumulate all the facts we
can, upon such abstruse points of patho-
logy as that now before us. It is on this
account we have given a brief analysis of
M. Pelletier's paper.?Revue Med. Nov,
1828] British Periodical Press. 441
BRITISH PERIODICAL PRESS.
x. ED. MED. & SURG. JOURNAL.
The race of medical periodicals cannot
boast of high pedigree. But what they
waat in genealogy they make up in fe-
cundity. There was a time, ('tis sixty
years since) when our grandfathers brought
forth much less frequently than our grand-
mothers?and when the sun paid a visit
to all the signs in the zodiac, daring each
term of literary pregnancy ! It was in
that peaceful period the dynasty in ques-
tion commenced, under the modest ap-
pellation of " MEDICAL COMMENTATORS"
~?for critics were not then known in me-
dical literature. The ostensible object
?f our forefathers was precisely what is
now alleged by their numerous progeny.
" A scheme, better calculated for saving
time in reading, and expense in purchas-
lng books, is a concise view of the books
themselves."?Introd. tovol. i. Jan. 1773.
Thus we see that our Caledonian ancestors
hud an eye to economy, even in those days.*
The dynasty of commentators lasted
20 years and upwards, when, without any
very apparent cause, it changed its title
to ANNALISTS, (ANNALS of MEDICINE) but
still pursued its slow annual phases, re-
turning with Christmas, or the new year,
to add to the enjoyments of those festive
epochs. The annals continued from
1796 to 1805, when an impulse was
evinced, that might well be deemed pro-
phetic of what is now taking place, after
a lapse of 20 years. Again the family
name was changed?the publication was
made quarterly, instead of annual, and
the Journal at the head of this article
started into existence.-^ It appears that,
even then, it was customary to declaim
against " the multitude of similar publi-
cations." Where are the records of this
multitude ? The following sentence will
explain. "All such periodical works,
from the very nature and origin of them,
have their periods of vigour and decay.
They flourish, and they fade. They ter-
minate, abruptly; or, after reaching a
respectable old age, they are suspended.
New ones arise. Their powers are ra-
pidly developed, &c." And, we may add,
they follow the fate of their predecessors,
sooner or later '. Gibbon tells us that, in
the Byzantine empire, although the grave
was dug at the very foot of the throne,
the latter never wanted a tenant, how-
ever soon he was destined to descend
into the gloomy habitation below ! The
dynasty of the Duncans has had a long
and honorable career. The three series,
spread over a space of half a centuiy,
have maintained a more uniform character,
dignity, and respectability, than any work
of the kind, in this or any other country.
The cause of this uniformity was, doubt-
less, owing to the government of the
journal being patriarchal, and, therefore,
not subjected to perpetual vicissitudes in
its editorial management. The innume-
rable contributions to this work varied
with the contributors?and although many
able and erudite reviews are scattered
through its volumes, we cannot say that
the work, as a whole, has evinced any
very powerful talent, or exuberant prin-
ciple of vitality. Its original conductors
have, we believe resigned?and how far
its present Editors may enhance, maintain,
or diminish the fame of this father of Bri-
tish periodicals, in an age when all the
energies of the human mind are called
forth, it is not for us to predicate.
No. 94.?Jan. 1st, 1828.
This Number of our Edinburgh cotem-
porary contains 13 original papers, occu-
pyingl 18 pages of the work, and evincing
various degrees of merit. The first on the
list is that which we shall notice in the
present fasciculus of our Journal.
Treatment of Caries. By Dr. J. J. Nicol.
After some desultory remarks on the;
* The Edinburgh Journal has sadly di-
verged from its original purpose. No
?ne now* expects any thing like an ac-
count of the medical literature of the day
ln the review department of that work.
t Let those who startle at changes in
the mode of publication, look back at the
history of periodicals, and they will find
changes enough. They will find that, in
almost every instance, the change has
been for the better, when the periods of
publication were shortened.
442 Periscope ; on, Circumspective Review. [Jan. 12
diseases of bone generally, Dr. N. comes
to the pith of the communication?the
treatment, by the application of lunar
caustic to the bone or its periosteum,
aided or not by the internal use of sarsa-
parilla, and general constitutional reme-
dies. Dr. N. modestly premises, that he
offers these cases to the profession, " with-
out the least pretensions to novelty, in
so far as regards the remedies employed."
What, then, it may be asked, was the
object of publication ? "The success of
their application alone has a claim on
its attention." Here the modesty is not
quite so striking as in the preceding sen-
tence. If the remedies are not novel, but
only the success of their application re-
markable, then the operator has the cre-
dit. Be this as it may, a single case will
illustrate the mode of treatment pursued
by our author, as well as a whole sheet
of didactic precepts.
" Case 2.?A young gentleman, about
16 years of age, having over-exerted him-
self in running, was seized with inflam-
mation along the right shin, extending
towards the knee. The surgeon who
attended leeched and applied evaporating
sedative lotions, by which the inflamma-
tory process was in a great measure sub-
dued. Ulceration however followed some
time after along the flat upper part of the
tibia; and the integuments gave way in
two places, about the distance of an inch
and a half from each other. The dis-
charge became limited and unhealthy;
the upper end of the tibia enlarged ; the
knee excessively pained; and the gene-
ral health bad. Leeches and evaporating
lotions were again applied over the knee
without any benefit, and diaphoretics
were also freely administered without
any relief. The case becoming alarm-
ingly retrograde, and the parents anx-
ious, I was called in. The knee was
much swollen, and the leech-bites had
become vesicated, presenting the appear-
ance of pemphigus. The whole upper
cud of the tibia, and for some inches
downwards, was evidently enlarged; its
anterior tubercle projected unusually, and
was soft, but not tender to the touch.
The orifices of the ulcerations previously
described had enlarged to nearly an inch
in diameter, communicating freely with
each other, and exposing the periosteum,
which was glairy, and discharged a thin
sero-gelatinous rather than sero-purulent
matter. Hectic had existed for some
time, and the strength was much ex-
hausted. The tibia was now pronounced
in a state of extensive disease internally,
and the treatment completely reversed.
An incision with a scalpel was made on
the outside of the enlarged tuberosity,
and a rod of caustic introduced close to
the bone. One single point of the peri-
osteum at each of the ulcerations below
was touched in a similar manner. Fo-
mentations of poppy capsule were applied
two or three times daily; four ounces of
Decoct. Sarsaparillce comp. were given
morning, noon, and night; and from half
a grain to a grain of opium exhibited two
hours after each dose of the decoction.
" In twenty-four hours tranquillity was
restored to the limb. In three days the
hectic fell off. This plan of treatment
was continued, substituting for the opium
as many tea-spoonfuls of the following
mixture as the stomach could bear with-
out sickness or nausea.
" Ant'im. tartarizat. gr. iv.
Solv. in aq. cinnam. ?viij.
Tine, opii, 3j- M."
The swelling of the knee disappeared
?that in the bone diminished?and two
thin circular films (evidently separations
from the outer lamina of the tibia) were
discharged at the openings. The ulcers
then turned healthy, and, in about four
months from the time Dr. N. first saw
the patient, a cure was effected, and the
young gentleman is now a useful limb of
the profession himself.
Dr. N. makes several judicious obser-
vations on individual remedies, employed
in these diseases of the bones and their
coverings. General blood-letting can
only be resorted to in the acute stages,
where the inflammation partakes of the
gouty or rheumatic character. Instead
of antimony, opium, and calomel, and
other remedies, formerly much employed,
our author now depends greatly on col-
chicum, with Battley's liq. opii sedativus.
These he uses in the proportion of two or
three parts of the former to one of the
latter?thus, giving to an adult 30 drops,
or more, (of the combination) every three
or four hours, according to the urgency
of the symptoms, continuing the medicine
till the stomach or bowels become af-
fected, or copious perspiration is excited.
^828] London Medical Repository. 443
hen, by these means, aided by sarsa-
parilla, and proper local treatment, the
disease has been arrested, but the bone
still continues enlarged and the perios-
teum thickened, then the establishment
?f a caustic issue becomes necessary.
c When the most prominent part can be
easily reached, I have invariably made
an incision with the point of a scalpel,
down to the bone, and not larger than
Was sufficient to admit a pointed rod of
lunar caustic, of the usual size, which
was immediately introduced, twirled round
two or three times, and instantly with-
drawn." A poultice was then applied,
till the eschar separated, and a purulent
discharge was established. Dr. N. affirms
that?" short of the actual excision of the
parts, he considers the lunar caustic to
-be the sheet anchor in the treatment of
^Sections of the bones."
We shall notice the other articles in
succession?at least all those which we
deem practically useful or theoretically
mteresting. And we take this oppor-
tunity of observing, that our Periscope
of Journals will always partake of the
character which we have endeavoured to
establish for our Analytical Review of
Books. We shall not give a bare cata-
logue?or a meagre skeleton of papers,
divested of blood, nerve, and flesh: Of
those articles which we do notice, we
shall aim at giving a concise, but intelli-
gible view of their import and bearing,
not a tantalizing glimpse, conveying no
definite ideas to the minds of our readers.
Let this be borne in mind.
2. THE LONDON MEDICAL
REPOSITORY.
This journal started on a flo'cFd-tide of
Popularity that promised to carry it to
immortality. It was presented to the
Public as " the Surgeon-Apothecaries'
and Apothecaries' Journal and Re-
view," in the year 1814, when a strong
excitement and impulse existed through-
out that wide and respectable class of
medical society, to distinguish itself by
science, art, and literature. The journal
was conducted by three eminent general
practitioners?Messrs. Burrows, Roys-
ton, and Thomson?so that every thing
conspired to the success of the work.
This success was great?but it was asto-
nishingly over-rated. The first number
circulated to the extent of 1250, and thi3
point was never afterwards over-stepped.
Four years later, (1818,) when thr pro-
perty was sold to Messrs. Underwood,
the circulation had dropped to 1000. One
of its professed objects was?" to bring
within the reach of the country practiti-
oner whatever of novelty arises in theory
or practice"?and to " assist in the ame-
lioration of medical literature, by inciting
to the practice of composition." In res-
pect to their review department, the
editors were inclined to think?" that
analysis which gives a faithful view of
facts to be more useful to the public,
than that criticism which aims to exalt
the critic above the author." Most peo-
ple are now of the same opinion.
The London Medical Repository has
passed through many hands during the
last ten years, and consequently taken its
hue and intellectual character from its
reigning master or masters.* While,
originally, the organ of the general
practitioners, it strenuously advocated
the interests of that body, but never in-
sulted any other class of medical society.
The consequence was, that it had the
support and respect of every designation
of the profession.
No. 31. January 1, 1S28.
The first article is the commencement
of an hospital report from Dr. Carter, of
the Kent and Canterbury Hospital. Dr. C.
informs us that, for several years, inter-
mittents were almost unknown in his
neighbourhood?even in Faversham and
the marshy lands of its vicinity. The
foul fiend was supposed to be extinct?
or to have taken his departure for Wal-
cheren or Batavia. But no such thing.
Within the last two years, ague ha3 re-
turned?prevailed?in many instances
been extremely obstinate?and, in some,
has laid the foundation for irremediable
disease. It is hardly necessary to say that
these effects of protracted ague are chiefly
felt in the liver and spleen, leading ulti-
* It has vacillated between aristocracy
and democracy?it has been on the verge
of free-thinking?and it has been down-
right methodistical.
444 Periscope; or, Circumspective Review. [Jan; 12
mately to dropsy. There is nothing new,
in the remedial way, brought forward by
Dr. Carter. The quinine and arsenic
were, of course, the most effectual reme-
dies for stopping the paroxysms?but
every relapse deteriorates the organs and
their functions, rendering it necessary to
give aperients, mercurials, bitters, and
vegetable tonics. We learn from Dr. C.
that his colleague, Dr. Chisholm, has
successfully practised the plan of Dr.
Mackintosh?bleeding in the cold stage.
Three fatal cases of organic disease of
the liver are detailed; but they present
only the usual phenomena of that dire
affection.
2. Phlegmonous Erysipelas. Mr. Evan
Daniel has published some observations
on the treatment of the above species of
erysipelas?"tending to shew the utility
of abstracting blood locally?against
which practice, most writers on this dis-
ease appear to be very much prejudiced."
Mr. D. we imagine, labours under a mis-
take. No modern writers or practitioners
are prejudiced against local bleeding in
phlegmonous erysipelas. It is in the
treatment of the superficial or cutaneous
erysipelas, that there is considerable dis-
crepancy of opinion. All parties are
agreed as to the utility of local depletion
in the phlegmonous form?but each has
his favourite mode of proceeding. Mr.
Lawrence makes a long cut, (he is a cut-
ting kind of chap at all times)?Mr. Hut-
chison makes short cuts?Mr. Travers
cuts both plans, and leaves the opera-
tion to leeches?Dr. Babbington and his
Greenwich friends make small cuts or
punctures with the point of a lancet.
These different parties have different
views of the modus sanandi of local de-
pletion. The long cutters believe that
it is of as great importance to take off
tension, as to empty the vessels of the
part?the leechers, lancers, and short
cutters evidently look to simple deple-
tion as the main benefit to be derived
from the measure. In this class Mr.
Daniel ranks himself?and this is the
whole of the business.
3. THE LONDON MEDICAL GAZETTE.
After a considerable note of preparation,
and with an address penned (if report be
true) by no less a personage than the
POET LAUREATE, the MEDICAL GAZETTE
has come forth, to be conducted on the
"ideal model" of "judgment, know-
ledge, and good feeling,"?in short,
to be the strenuous advocate of philo-
sophic views and liberal sentiments. For
these good intentions we give our co-
temporary full credit ; and, as far as it
is concerned in the laudable endeavour to
counteract the baleful influence of the
organ of defamation, it will find in us a
zealous, though a very humble auxiliary.
As there appears to be a greater leaning,
however, in the Medical Gazette, to-
wards " things that be," than comports
precisely with our sentiments, so, it is
not impossible that we may have some
little collisions in medical politics. In
our differences, however, with the Medi-
cal Gazette, we hope to conduct our-
selves with proper attention to that "ideal
model," which our cotemporary has judi-
ciouslyplaced before its eyes, for constant
imitation. The Lancet has now got its
match in the field. For some time we
feared the Medical Gazette would wrap
itself too much up in its own dignity, and
thereby prove tame. It seems to have
taken the hint thrown out in our last
number. It is now shewing spirit, and
we predict that the reign of the Lancet
is overl The Hospital Reports, consti-
tuting the chief value of all weekly publi-
cations, are given in the Medical Gazette
infinitely better than in the Lancet, with
the incalculable advantage of being true.
Med. Gazette, Nos. 4 and 5.
clinical lectures.
The first article in the 4th number of
our new cotemporary is the substance of
a clinical lecture by Mr. Charles Bell, on
diseases and accidents of the hip-joint.
We may take this opportunity of express-
ing our opinion, that clinical lectures are
the only proper lectures for reporting in
a medical Journal. They are, like the
debates in a medical society, mere verbal
comments on a given subject, which
would be lost, if not recorded, and which
may be serviceable if noted faithfully and
published correctly. Not so with elemen-
tary lectures that are constructed for the
purpose of annual delivery. These are
1828]
The London Medical Gazette. 445
as much a man's private property as the
coat on his back, 01* the watch in his fob :
and whoever publishes without licence
such lectures, is guilty of literary spolia-
tion, according to the laws of honour and
conscience, however incognizable such
?in act may be by the laws of the land.
For the truth of this sentiment, we ap-
peal to the breast of every unprejudiced
Bian in the profession.
But, to our business. Mr. Bell, after
referring to five or six cases in the wards
?f the Middlesex Hospital, presenting
various specimens of hip-disease?some
with the leg of the affected side longer
than the other?some with it shorter?
some appearing to be on the point of
anchylosis?and two in a state which
rendered it doubtful whether there was
fracture or dislocation of the femur?
proceeded to make some general obser-
vations on hip-diseases.
This affection is common to infancy as-
Well as age. Mr. B. illustrates its occur-
rence in the former state, as follows :?
an infant was suddenly seized with con-
vulsions, and the fits returned again and
again, so that the child was considered
to be in great danger. Dr. Denman
thought it was owing to dentition, and
requested Mr. B. to lance the gums deeply
and completely round the jaw. He did
so, but no benefit resulted. On careful
examination of the naked infant, the
disease was found to be in the hip, which
Was much swelled, tender, and painful
motion. The disease went on, and
suppuration surrounded the whole joint.
Punctures were often made, and, after a
long illness, the little patient finally re-
covered, though he is lame, from wasting
?f the head of the femur.
There is another way in which the dis-
ease may present itself. A boy at school
is observed to limp, and, in the evening,
complains of pain and stiffness. He re-
turns to his exercises, and when warmed
by these, his lameness and stiffness dis-
appear. Still in the evenings he com-
plains?and, after a time, he is observed
to hobble more in his gait. He will now
probably acknowledge that he suffers a
good deal in the limb?and now it is that
^edical advice is sought. The examina-
tion is to be conducted in the following
banner:?
" The first thing you are to do is to
make him walk before you, and observe
the direction of the toe. You are then
to strip him, and place him with the but-
tocks directly before you. You will ob-
serve that on the affected side there is a
fulness and a greater breadth betwixt the
fissure of the nates and the trochanter
major than on the other side ; and if
the trochanter be firmly pressed inward
against the acetabulum, pain will be felt.
The patient may in the next place be
made to stand erect on both his legs ;
you then ask him to rest upon the sound
limb only, and throw cut the other as iii
abduction. This you will probably find
he cannot do without great pain ; be-
cause, during this movement of the limb,
the muscles press the hip-joint, and the
head of the bone jars against the delicate
and inflamed structure of the acetabulum.
If he be now laid straight upon his back
on the carpet, you will find an inequality
in the length of his limbs. If the patient
and others of the family exhibit the pecu-
liar signs of a strumous diathesis, then
there is every probability of this being
the commencing stage of that very dan-
gerous disease, which is called by authors
Morbus Coxarius."
Another boy returns from school, and
is observed by his mother to be lame or
awkward in his gait. He is not conscious
of it, and affirms that he played a match
of cricket yesterday. On examination,
the surgeon perceives that the boy walks
lame?yet he can throw the leg about in
every direction, without pain. On laying
him prostrate on the floor, one leg ap-
pears shorter than the other. The short
leg will also be found smaller than the
other. This depends on scrofula. At
a certain period, the boy's leg stops
growing, a frequent occurrence during
dentition, when the bowels are much dis-
ordered. This affection is liable to be
confounded with disease of the hip, but it
is totally different. Leeches, blisters,
and the usual means, are here sometimes
applied?with no good, but, probably,
bad effects.
In certain cases, (one of which was
then in the wards,) the child complains
solely of the knee, a common occurrence
in disease of the hip-joint, owing, Mr. B.
thinks, to inflammation having spread to
the obturator nerve, as it passes through
the thyroid foramen. The pain is felt in
the branches of the nerve, according to
a general neurological law. In such
446 Periscope; or, Circumspective Reviett. [Jan. 12
cases, when pressure and kneading of the
knee occasion no pain, we may suspect
the hip-joint to be the seat of the disease.
In respect to shortening of the litnb,
Mr. B. makes many excellent observa-
tions, and we have quoted a passage from
this part of his lecture, when speaking
of a paper on supposed fractures of the
cervix femoris, within the capsule, by Mr.
Langstaff. When the disease commences
within the joint, it extends its influence
to the surrounding parts, which become
inflamed, and abscesses form. The pa-
tient lies with the body inclined forward,
and the knee raised towards the belly.
In this way he twists the spine, and in-
clines the pelvis, drawing it obliquely
upwards on the affected side. This ap-
parent shortening of the limb is some-
times erroneously attributed to absorp-
tion of the neck of the bone. In the
case before him, the limb, on the diseased
side, was longer than the other?yet still
it was owing to twisting of the pelvis.
Hut the apparent shortening of the limb
is the common occurrence, and if this be
perceived on the second or third day after
the commencement of acute inflamma-
tion of the hip, the surgeon may begin to
fear that he had made a mistake?that
there had been a fracture or dislocation
of the femur that had not been detected.
" In order to satisfy yourselves whe-
ther there is real, or only apparent short-
ening of the limb, make the patient lie
even upon his back, in as straight a po-
sition as he can, and then search for the
superior spinous processes of the ilium
on both sides. By drawing a tape be-
tween these two points, it will be seen
whether the pelvis is situated obliquely
or not. If the pelvis be placed exactly
in a straight position, then the difference
in the length of the two limbs no longer
exists : the heels may be made to meet
and correspond exactly. But whenever
the examination is over, the patient gra-
dually resumes his former position of ease,
and then you find that the limb is drawn
upwards, and appears shortened just as
before."
A case in elucidation is given, and also
a wood-cut, shewing the alteration in the
head and neck of the femur produced by
inflammation. This clinical lecture is
creditable to Mr. Bell, and affords him
opportunities of shewing his ingenuity in
the explanation of morbid phenomena
connected with the mechanism of the hu-
man frame.
4. THE MEDICAL AND PHYSICAL
JOURNAL.
This Journal originated in cow-pox, in
the last year of the last century. Its
success was far beyond that of any other
medical journal that had previously ap-
peared. It became nearly as epidemic
as vaccination. We have seen documents,
in the possession of the late Mr. Thorne,
the original printer of the journal, prov-
ing that its circulation, for some years,
amounted to nearly 3000 copies per month!
It will soon have attained its 60th volume
?a grand climacteric in periodical lite-
rature ! A list of its editors would nearly
cover a page of our work?and the vari-
ous tints and hues which the Medical and
Physical Journal received from its suc-
cessive directors, are far beyond our pow-
er of painting. We wish it every success.
The Number for Jan. (19, New Series)
opens with a paper on the physiology of
the circulation, by Mr. James, of Exeter;
in which nothing new is advanced, and
nothing old is established or refuted.
Mr. James seems to come to the conclu-
sion, (to which we long ago came, and
which we have repeatedly published,) that
the blood does not go along the arteries
in waves, but that, at each ventricular
contraction, " a shock is diffused over
the whole arterial system, which propels
the blood in a longitudinal direction."
There is, no doubt, a very trilling instan-
taneous dilatation of the whole tree of
arteries at the same time, the recoil of
which feeds the capillary system till the
next ejection of blood from the heart.
Mr. James affects to treat the suction-
influence of the chest and heart as chi-
meras of the imagination. It is needless
to say, that he offers nothing like facts
to substantiate this negation.
In the second article, Sir George Gibbes
continues his speculations on life, on ani-
malcula, on monades, on ultimate living
particles, terminating rudiments, &c.
through which speculations we dare not
follow him. Sir George appears to be
enamoured of the following " very novel
and most curious" doctrine.
" All the tissues of the animal body are
shewn to be ultimately resolvable into
1828] Lancet. 447
Qunute globules, and that these globules,
3s they are successively disengaged from
the mass, exhibit distinctly a power of
spontaneous activity, moving about ra-
pidly in an directions. In short, they
become animalcula, possessing the power
locomotion, and have been named mo-
nades. It appears that these bodies are
capable of existing as animals or vegeta-
bles, and of forming elementary parts of
either. Thus, according to the above
view of the subject, we arrive at the sin-
gular conclusion, that the human body,
with all its organs, is built up of animal-
cula, and that it is a congeries of count-
less millions of organized beings, each
capable of living in a separate state, and,
perhaps, exercising some of the functions
?f individual life, whilst incorporated with
?ur system. It is not certain, but it is
at least probable, that these monades form
the last link in the chain of organic life,
and that, beyond them, there is nothing
but the ultimate gazeous elements."
The author of this curious theory be-
lieves the process of digestion to consist
Merely in the operations necessary to
Separate these monades from the combi-
bations in which they existed in the ani-
mal and vegetable substances that form
?ur food, while assimilation is the mere
transport of the monades to the various
Parts of the body.
How will Sir Gilbert Blane, Professor
?1-ande, and Doctor Reece chuckle, when
they peruse this theory, so beautifully cor-
roborative of their doctrine, that Thames
Water is both wholesome and fattening !
^o wonder that it is so, when it contains
so many millions of animalcula, all ready
disengaged to fly to the different parts of
the body, without any digestive process
being necessary!
Vaccination of the Sultan's Children.
Baron, of Gloucester, has transmitted
the important information, that three of
the Grand Seigneur's children have un-
dergone vaccination. It appears that
His Turkish Majesty has been inoculated
With the contagion of European discipline,
ever since the destruction of the Janis-
saries. Nothing is now heard, in Con-
stantinople, but military music?nothing
played except European airs?nothing
seen but drum-majors, great canes, mus-
kets and bayonets?and the Grand Seig-
neur himself, in a General's uniform,
ordering manoeuvres ! This fine flourish
introduces us to the important intelli-
gence, that Dr. Auban has had the honour
of vaccinating three of the Sultan's chil-
dren, in order to complete the revolution
that has been effected among the follow-
ers of the Prophet.
Paraplegia. Dr. Thomas, of Devon-
port, has detailed a case of this kind,
which occurred in the person of a sailor,
aged 43 years. He had suffered an at-
tack of fever fifteen years previously in
India, and one of acute rheumatism in
the Winter of 1824. After sleeping in a
damp room last Spring, he felt pain in
the left side, extending to the back. This
was followed by weakness and numbness
in the lower extremities, constipation of
bowels, dysury, soon amounting to reten-
tion. There was no affection of the head.
We need not detail the treatment. For a
time the patient improved?but after-
wards he became completely hemiplegias
Ulceration took place over the sacrum,
and then the paralysis of the lower ex-
tremities diminished?till it might be
said to be almost gone. Ulceration of
the bladder, however, occurred, and of
this the patient died. No dissection could
be obtained. The head remained free
from disorder all the time.
Dr. Thomas seems to have brought
forward this case, with the view of as-
sisting Dr. Burder in refuting the doc-
trine of the late Dr. Baillie, that para-
plegia generally depends on affection of
the brain. But there is hardly any one
who maintains such a doctrine now?and
the case of Dr. Thomas is defective, from
want of dissection. We have no doubt,
however, that, in the above case, the dis-
ease was in the spinal marrow, and that
the ulcer over the sacrum acted the sa-
lutary part of a caustic issue.
The hospital reports, in the Medical
and Physical Journal we shall amalgamate
with others under their proper heads.
5. LANCET. JANUARY 5, 1828.
It is not our intention, except on very
extraordinary occasions, to notice the
elementary lectures in the columns of the
Lancet. They are calculated for two
classes of readers?those who have lost
448 Periscope; or, Circumspective Review. [Jan. 12
their dentes sapientiae from old age?and
those striplings who have not yet cut
them. The lectures, indeed, of a Cooper,
an Abernethy, and a few others, did pos-
sess interest?but now?we have had
satis superque. The clinical lectures, as
we have elsewhere observed, are the pro-
per subjects for reporting.
In this Number are given some clinical
remarks, by Dr. Elliotson, on chronic af-
fections of the brain. The subjects in the
hospital were chiefly males, between the
age jof 25 and 40?the causes, in many
instances, were external injuries. The
symptoms were, generally, pain in the
head, drowsiness, vertigo, throbbing, di-
lated pupils, tinnitus aurium, &c.?in
short, the symptoms of chronic inflam-
mation?and the terminations were too
often in hemiplegia, epilepsy, convul-
sions, &c. The post-mortem appear-
ances, in Dr. E.'s practice, were those
which others have observed?effusion,
thickening of membranes, ramollisse-
ment, &c. The treatment may be very
easily anticipated, ? spare diet, ? open
bowels?cold, repeated blisters, cupping
and leeching ("almost endlessly") to the
head ? abstraction of blood from the
hypochondria or epigastrium?mercury,
which should never be omitted. A case
was related, and a brain exhibited where
there was serous fluid found on the sur-
face of the brain, as well as in the ven-
tricles?also a quantity of limpid fluid on
the medulla spinalis. The reporter makes
Dr. Elliotson to say that the case in ques-
tion is one not only instructive in itself
but as " mutilating" against the opinion
advanced by Majendie, that the fluid
found in the ventricles comes from the
spine entirely, or in the greater part;
and, consequently, that our remedial mea-
sures should be chiefly directed to that
region. We do not deem it necessary to
enter into the investigation here, whe-
ther or not there is a communication
between the ventricles of the brain and
the spinal canal.
Cooper versus Lawrence.
Cantaie pares, et recantare parati.
There is, at this moment, a tremen-
dous conflict between two surgical instru-
ments?the trephine and the scalpel?
the Lancet being, of course, the bottle-
holder. Mr. Lawrence appears to have
thought that there would not be sufficient
space on Mr. Cooper's head for a 14 inch
incision?and therefore he has made seve-
ral perforations to get at the spinous
artery of his antagonist. Mr. Cooper, on
the other hand, has taken up the scalpel,
and, avoiding all attempts to get at the clura
mater of Mr. Lawrence, amuses himself
by making short, but pretty numerous in-
cisions into the soft parts of the enemy.
How this deadly contest will end we can-
not yet predict. Both parties appear at
home in the use of their weapons, and, it
is to be feared, that blood will be spilt
and bones broken before the victory is
proclaimed.
Duel.
We have now to record a much more
serious kind of fighting than that with the
scalpel and trephine. A duel was fought,
on Saturday week, between Dr. Forbes,
of Argyll-street, and Mr. Thompson, a
young surgeon, a pupil at the Eye-Infir-
mary, to which Dr. Forbes is physician.
No blood was shed, but a ball went through
Mr. Thompson's hat, when the secQnds
interfered. The circumstances that led
to this meeting are pretty well known.
Very late in the evening, before Mr. Guth-
rie's action was to have come on against
the Lancet, Dr. Forbes wrote to Mr.
Guthrie, intimating that, if interrogated
in court, he would be forced to condemn
certain parts of Mr. Guthrie's practice,
or words to that eft'ect. This, of course,
stopped proceedings in court next day,
and the business, we suppose, is ended
for ever. It seems that Mr. Thompson
was indignant on this occasion, and in-
sulted Dr. Forbes?hence the duel. We
join with our cotemporary in considering
the above as a striking illustration of the
tumultuous state of our profession, " It
is one among the daily proofs of the in-
calculable mischief resulting from that
system of depravity in the medical press,
which has thus literally set man in hos-
tility to man, for the profit of a
." We dare not close the sen-
tence from our cotemporary, lest we should
have another judicial action on our hands!
We confidently believe that the " system
of depravity" is beginning to totter?and
we hope to give it a shove before the end
of the year 182S.
1S28] Hospital Practice. 449
HOSPITAL PRACTICE.
Under this head, we hope to give a selec-
tion and re-union of important cases re-
ported from the public institutions of this
and other countries, which will prove the
?iost valuable department of a journal
ever offered to the medical practitioner,
?f town or country. We shall take great
care to disembarrass reports of all useless
?r irrelevant verbiage?nor shall we no-
tice those cases that afford no useful prac-
tical hints, or interesting phenomena. In
our criticisms, we shall be guided solely
by rigid impartiality?and a desire to cor-
rect error and establish truth.
1. ST. GEORGE'S HOSPITAL.
State of the Pupil in deep Intoxi-
cation.*
A. young man was brought into this hos-
pital, some little time ago, having fallen
from the Bristol coach half an hour pre-
viously, in a state of complete intoxi-
cation. When admitted, he was quite
insensible?the pulse 70, and rather la-
bouring?the pupil dilated at first, but,
in the course of a very short time, it
had become contracted almost to a pin's
Point. At the back of the head there
Was a circular wound of the scalp, about
the size of a shilling, exposing the bone
perfectly denuded beneath, but unaccom-
panied with fracture or depression. In
tlie course of the day the pulse got up,
a?d he was bled; and in the evening he
brought up, by vomiting, a most disgust-
ing mass of pork, brandy, &c. after which
be became much more sensible, and the
pupils began to act. Next day he com-
plained of nothing but the scalp-wound,
in spite of which, and all Mr. Rose's
remonstrances to boot, he walked out of
the hospital, got upon the top of the
coach, and set off once more for Bristol.
Upon this case the reporter remarks?
" We mention it principally with a view
?f calling the attention of our readers
to the excessive contraction of the pupil,
a state which Mr. Rose says he has gene-
rally seen in complete intoxication. In
this patient there were many of the symp-
toms of compression of the brain; and
indeed there cannot be a doubt that indi-
viduals are occasionally most severely,
and even fatally treated for such com-
pression, when rest, or, it may be, the
stomach-pump, are all that is wanted."
Strangulated Hernia.
Two cases are reported in the Medical
Gazette, (No. 4,) which we shall notice,
in order to shew Mr. Brodie's practice.
The first case was that of a stout young
countryman, admitted under the care of
Mr. Rose. The hernia, had existed since
childhood, was never completely redu-
cible, and the symptoms of strangulation
had obtained for twenty-four hours prior
to the performance of the operation. On
opening the sac, a knuckle of intestine
was found, which was returned, but, be-
sides this, there remained a quantity of
omentum, which had contracted old and
strong adhesions to the sac. This was
left unreduced; an abscess subsequently
formed in it, attended with pretty urgent
symptoms, but under the judicious treat-
ment of Mr. Rose the patient did remark-
ably well.
The second case occurred toMr.Brodie.
The patient was a watchman?the symp-
toms commenced at 5, a.m. of the 15th
December, and the operation was per-
formed at 3, a. m. of the 16th. As in the
former instance, intestine and omentum
were down, and the circumstances being
the same, the intestine was returned, and
the omentum left undisturbed in the sac.
This patient also did well.
Surgeons are now so universally im-
pressed with the advantages of the early
operation in hernia, that we shall say not
a word about that. The treatment of the
omentum in these cases is the point to
which we would wish to direct the atten-
tion of our readers. Sir Astley Cooper,
in his published lectures, (and a greater
boon than these lectures, we think, has
not been conferred on the rising profes-
sional generation for these many years,)
makes the following remarks. If the
omentum be very large?mortified?or, if
it be " hardened and have a scirrhous
feel," it should be removed wholly, or in
part, the divided vessels secured by fine
ligatures, and these, one end being cut off-,
3 E
* London Medical Gazette, No. V
Vdl. VIII. No. 16.
450 Periscope; or, Circumspective Review. [Jan. 12
should be left hanging from the wound.
If omentum alone adheres to the sac, it
may be freely separated and returned. So
far, Sir Astley; Mr. Brodie, however,
thinks otherwise, and as the reporter has
detailed his method of treatment, we shall
take the liberty of quoting the passage,
without note or comment of our own.
" In his (Mr. Brodie's) Clinical Lec-
ture upon Joseph Haplin's case, he ob-
served, that whenever difficulty obtained
in reducing the omentum, either from
its being adherent, mortified, or from its
being converted into a large fatty mass,
the old practice used to be to cut it off,
or apply a ligature upon it. With regard
to the ligature, authors are almost una-
nimous in disapproving of it; in fact, as
Sir A. Cooper shrewdly remarks, it is
renewing with increased severity what
the operation was intended to relieve?
the stricture on the part.
" Mr. Brodie is inclined to think that
the excision of the omentum is not so
devoid of difficulty and danger as some
would seem to infer. If no vessels are
tied, the hemorrhage may be very con-
siderable ; indeed, Mr. Hey has pub-
lished two cases in which the patients
nearly died of it. If, again, the arteries
are tied, the ligatures cut off close, and
the omentum returned, we are not sure
that bleeding may not take place within
the abdomen, from vessels which did
not bleed while the omentum lay ex-
posed in the sac, and which therefore
were not secured : and, besides, what is
to become of the ligatures? Mr. Earle
related to Mr. Brodie a case of this kind,
in which they were discharged through
an abscess which presented itself at or
near the umbilicus. The patient reco-
vered ; but, nevertheless, an abscess of
the belly, which bursts at the umbilicus,
cannot be supposed to be always free
from danger. If, instead of the ligatures
being cut close, one end of each of them
be left hanging out at the neck of the
hernial sac, the omentum must of course
remain drawn down to the abdominal
ring ; and in what respect is the patient
better off than if a portion of it had been
left in the sac itself? There is also some
danger in this case of inflammation of
the cut end of the omentum, and Mr.
Brodie mentioned one case in which an
abscess formed in the omentum, at this .
part, immediately within the internal ab-
dominal ring, and the patient died in
consequence.
" He, however, stated that he did not
mean to condemn the excision of the
omentum in all cases, but merely to ex-
press that it ought not to be done without
a very sufficient reason ; and that in
cases where there is not a very large
quantity of omentum in the hernia, but
where it is extensively adherent to the
surface of the sac, it is safer to leave it
where it is found, than to cut off a por-
tion of it, or to dissect through the ad-
hesions. The success of this practice,
in the two preceding cases, is certainly
in its favour. The patients arc left as
well off as they were before the stran-
gulation took place ; and probably bet-
ter, inasmuch as it is most likely that
the omentum, after the operation, must
have contracted adhesions to the neck
of the hernial sac, making them less
liable to a descent of the intestine.".
2. MIDDLESEX HOSPITAL.
Anomalous CEdematode Swelling of
the Leg.*
A watchman, set. 32, was admitted in
October, with an enlargement in the left
leg and thigh, which had begun sud-
denly, as we understand it, three years
previously, had never diminished, except
when in the recumbent position, and was
productive of much distress and suffering.
His constitution was broken down?se-
cretions imperfect, and he voided with
much difficulty turbid and scanty urine.
The left leg was about 22 inches in cir-
cumference, natural in colour, tender to
the touch, elastic, but indenting on firm
pressure. He took diuretics, was cupped
and blistered for the first three weeks
after his admission,when an acute attack
of hepatitis came on, which was sub-
dued only by the most active treatment.
During the attack, the leg was free from
pain, and had nearly regained its natural
size, but the moment the hepatic inflam-
mation ceased, the swelling, &c. of the
leg returned. This happened a second
time in November, on the 25th of which
he left the hospital, in consequence of
some expressions inadvertently used, and
has never since returned. The reporter
* London Med. Gazette, No. V.
^828] Strangulated Hernia. 451
observes, that this case bears some re-
semblance to phlegmatia dolens, and in
a female, after parturition, might have
keen so considered. The symptoms,
however, of phlegmatia dolens do not re-
gain unrelieved for so long a period as
three years, though, certainly a degree
?f swelling of the limb may remain for
Me. To us, it appears that the case was
??e of chronic inflammation of the cel-
lular tissue generally, and this opinion is
strengthened by thefactofits alternating
u'ith the acute inflammation of the liver.
^V'e have seen one or two cases, some-
what similar, occurring in patients who
Were much exposed to damp and cold,
and where the appearance of the limb was
far
from being unlike that presented in
phlegmatia dolens, although the disease
ran on to a different and fatal termination.
3. ST. THOMAS'S HOSPITAL.
Auscess between the Bladder and
PuBES.
This case is related in the fifth number of
the Gazette. The patient was a female,
;et. 42, who had been married at the age
?f 20, and miscarried in the sixth month.
She was not pregnant again for 18 years,
when she again miscarried in the fifth
month, After this she did not menstru-
ate fur 18 months, when she was attacked
oy profuse flooding, which lasted for three
days. In the following month she began
to suffer from pain in the uterine and
lumbar regions, and November 21st she
entered the hospital under the care of
Di". Elliotson. She was sallow, debili-
tated, and complained of violent pain in
the hypogastrium, pubes, and loins, with
numbness and tingling in the right thigh.
Tenderness of the hypogastrium and pubes
to the touch?copious discharge of puri-
form matter from the vagina, occasion-
ally tinged with blood?urine generally
fi'ee, sometimes retained for two days to-
gether?general ill health. The uterus,
examination, was healthy, but the dis-
charge dark and fetid. These symptoms
were not relieved by calomel, leeches,
blisters, &c. &c.?the discharge conti-
nued copions?cough came on?she grew
Weaker, and on the Kith December she
sank.
' On examination, there was found in
front and behind the symphysis, extend-
ing beyond the external abdominal rings,
so that the round ligaments passed through
it, an abscess, containing dark and fetid
pus, which had a free exit through the
urethra. A portion of this was destroyed
?the surface of the bone was rough and
blackened, but not carious, whilst the
bladder nnd vagina were perfectly healthy.
Strangulated Hernia.
A case of strangulated hernia, terminating
fatally, is reported in the last Number of
the Lancet, which we deem it right to
notice. The patient was a stout man,
who had laboured under constipation of
the bowels for some days, attended with
pain and colic, for which, in fact, he was
sent into the hospital, no allusion being
made to hernia, which, however, was
speedily discovered there. The swelling
took the usual course of inguinal hernia?
occupied a portion of scrotum?and the
integuments were inflamed. The man
had vomiting and hiccup?the counte-
nance was not depressed?pulse feeble?
much tenderness of abdomen. He had,
according to his own account, a swelling
in the part since his birth, but latterly it
got larger. The taxis was ineffectually
employed, and then Mr. Green proceeded
to operate. The hernial sac contained
much serum, and a knuckle of intestine,
of a chocolate colour, was found, par-
tially adherent to the sac. The hernia
was of the congenital kind. Although
the ring was freely divided, a stricture
still existed, beyond the ring, girting the
intestine. This being cut through, the
gut was readily returned. It is the prac-
tice of Mr. Green not to give any pur-
gative medicine for some hours after an
operation for hernia, and this practice
was followed in the present instance.
The reporter, however, seems to have
discovered a mighty flaw in the Gazette
account of the case?namely, the admi-
nistration of a couple of glysters imme-
diately after the operation, instead of
some sulphate of magnesia the next day
at noon ! Most important mis-statement!
This beats the Lancet hollow. That ve-
racious instrument never committed such
an error, wilfully or accidentally ! But,
allowing that the enemata had been ad-
ministered immediately after the opera-
tion, it would rot have been an infraction
of the precept laid down by Cline, and
452 Periscope; or, Circumspective Review. [Jan. 12
followed by Green?that of not disturbing
the bowels too soon by purgative medi-
cine. The enema merely empties the
colon, and does not affect the small intes-
tine. But this is a piece of knowledge
far beyond the scope of the reporter's
eensorium. The patient died in three
days after the operation, and no dissection
was permitted. This case is spun out
with the most tiresome verbiage in the
Lancet, to the extent of four columns !
Surely some of the reporters must be paid
by the acre rather than by the sheet of
letter-press which they blacken in every
volume of the Lancet !
A. ST. BARTHOLOMEW'S HOSPITAL.
Fracture of the fourth and fifth
Cervical Vertebr/e.*
We cannot but congratulate our contem-
porary on his Hospital Reports. They
seem to be given in a spirit of great can-
dour and fairness, and the cases are ge-
nerally interesting, whilst they are unal-
loyed by that sickening ribaldry and slang
which grace this department of the Lan-
cet. This is as it should be.
Case. G. F. set. 45, was admitted un-
der the care of Mr. Vincent, at 8, a.m.
Dec 18th, having fallen on the back of
his head from the top of a loaded waggon,
by which he was stunned for the time.
On admission, the occipital bone was found
denuded of its pericranium for some ex-
tent?pulse small and feeble?sensibility
and motion diminished in the upper and
lower extremities?pupils contracted?
priapism?respiration chiefly diaphragma-
tic. When Mr. V. saw him at half-past
twelve, there was complete paralysis of
the lower half of the body, with pain in
the back of the neck, where a projection
could be felt, in the situation of the fifth
cervical vertebra. The pulse rose, and
at 12, p.m. he was bled to ?xvij. Next
day, the paralysis of the upper extremi-
ties had increased; he passed his stools
involuntarily, and the bladder required
the introduction of the catheter night and
morning. He was bled and purged, but
without any amendment, and on the 21st
he died.
Dissection. The base of the spinous
process of the fourth cervical vertebra was
fractured, and its right articulating pro-
cess thrown forwards on the fifth, the
body of which was completely fractured
through at its upper part. Blood was
effused between the vertebra; and the
theca, which was not torn, whilst the
chord in the situation of the fracture was
much swollen and softened, but not lace-
rated. Slight vascularity about the neck
of the bladder.
Of what use would trephining have been
here ?
Case 2?Morbid Growth in the Fe-
mur. The patient was a wretched-looking
creature, about 50, a martyr to rheumatic
gout. The disease in the knee was of
three years' standing, and had been once
almost, cured by an issue. The joint was
greatly swollen, and projected in a coni-
cal form towards the inside of the leg.
The apex appeared to contain fluid, but
the rest of the tumour was firm and un-
yielding, like a fungous growth. There
was excessive pain and constitutional dis-
turbance ; and, besides this, the poor
wretch had diseased bladder and stricture
of the urethra, so that he was no very
promising subject for an operation. This
was done by Mr. Vincent on a Saturday,
and, on the succeeding Friday, the pa-
tient had a fit of the gout, and died.
On examining the tumour, much chalky
substance was found in the subcutaneous
cellular tissue, and on the head of the fe-
mur, the internal condyle of which was
filled with a gelatinous, fungoid substance.
It was something like soft soap, had brok-
en down the bony walls of the condyle,
and made its way into the joint and its
neighbourhood.
It is not improbable, from the chalky
deposition in the joint and around it, that
this anomalous disease had its origin in
the gouty or rheumatic inflammation.
Hernia?Adherent Epiplocele.
The Lancet, for reasons best known
to itself, though, perhaps, obvious enough
to some others, has hunted back to the
month of November, 182(J, for a case ex-
emplifying certain points of practice, lately
reported from St. George's Hospital, in a
cotemporary journal. The object seems
to be, to shew that Mr. Lawrence is not
exactly of Mr. Brodie's way of thinking,
* London Med. Gaz. No. V.
1828]
Laceration of thb Urethra, ?S^c. 453
111 regard to the treatment of adherent
omentum. Non nobis inter vos, &c.
The patient was 60 years of age, and
had been ruptured for the last thirty. He
had worn a truss, though the reduction
^as only partial. After some unusual
exertion, on the 2d of November, a fur-
ther protrusion occurred?the parts be-
came painful?a surgeon tried ineffec-
tually to replace the rupture?and he
Was sent into Bartholomew's. He evi-
dently had strangulated hernia, and all
the usual means were pursued, (and mi-
nutely detailed, for the ten-thousandth
time,) without success. Mr. Lawrence
operated at the expiration of eight hours
from the new descent. Every stroke of
the scalpel is, of course, described?but
We shall inform our readers, at once, that
six inches of incarcerated small intestine
Were found, of a dark chocolate colour.
he stricture having been divided, an
indentation (most wonderful to relate)
Was found in thestrictured gut?and when
this was drawn down, a small opening
Was found in the intestine, just above the
indentation. " Mr. Lawrence supposed
that the bowel had been wounded by the
curved bistoury." He tied a silk thread
1-ound the aperture?cut away the ends
"?dissected away a portion of old, adherent
omentum?tied the vessels?andthewhole
Was then dressed lightly. The reporter
?f this case (which occupies four deadly
columns of the Lancet) does not say whe-
ther the intestine was returned into the
abdomen after ligature of the perforation.
The patient recovered without any bad
symptoms. Under the head of " Re-
marks," the reporter, who seems to have
an eye to the " main chance," in the
length of his cases, as well as his cuts,
repeats the whole business over again,
with one or two quotations from Hey,
Desault, Beyer, (a new name in the sur-
gical horizon) Lawrence, and Travers.
The whole case is unquestionably taken
from the porte-fcuille of a man of letters,
f?r a special purpose, which we need not
explain.
If ever a critical reviewer and condenser
Was needed in medical literature, it is at
the present moment, for hospital reports.
The writers are paid by the sheet?and
it is their interest to dilate?the critical
condenser is paid in a diametrically op-
posite manner?and the consequences are
obvious.
a. GUY'S HOSPITAL.
Laceration of the Urethra?Slough-
ing?Fatal Result.*
W. S. act. 14, was admitted, Nov. 19th,
under the care of Mr. Key, having fallen
on the perinaeum across an iron chain.
Severe pain and inability to make water,
followed the accident, and, in half an
hour, the scrotum began to swell, and
became red. On admission, the scrotum
was greatly distended and ecchymosed on
its posterior part, with slight effusion
into the prepuce.
Scarifications into the scrotum?-fomen-
tations?catheter to be kept in the
bladder.
22d. Had an exacerbation of fever last
night, but it is mitigated this morning??
ecchymosed part of the scrotum livid?
cellular membrane, which has been sca-
rified, dark and sloughy?yellow hue on
the lower part of the abdomen.
Quin. sulph. gr. jss. Camph. gr. ij.
Amm. carb. gr. x. 6tis horis?Cat.
cerevisicc.
Hiccough and tenderness of the hypo-
gastrium came on, and this was so severe
on the 24th that, to prevent infiltration,
it was determined to cut into the urethra,
pass a catheter through the opening, and
confine it in the bladder. On doing this,
it was found that two inches of the ure-
thra, anterior to the triangular ligament,
had sloughed. Bowels relaxed?pulse
130 and weak.
Tinct. opii, ff\ x. ex Julep, amnion.?
Arrow-root and brandy.?Quinine to
be omitted.
A slough formed at the back of the
scrotum, which separated and exposed
the tunica vaginalis of the right testicle.
On the 6th Dec. an incision was made over
Poupart's ligament, and a quantity of fetid
pus discharged, but, on the 15th, the boy
died. No dissection was permitted.
Mr. Key, in his remarks on this case,
observed that, in common cases of rup-
tured urethra, leaving a catheter in the
bladder generally suffices, the inflam-
mation arising from the extravasation
generally setting bounds to the escape of
the urine beyond the cells of the scrotum.
In this case, the infiltration was exceed-
ingly diffused, and free incisions into the
? London Med. Gazette, No. V.
454 Periscope; on, Circumspective Review. [Jan. 12
scrotum did not prevent the occurrence
of extensive sloughing, which, however,
was the immediate consequence of the
blow, and not of the extravasation. On
the 24th, when the pain and redness
about the pubes indicated still further
extravasation, a free incision in perineo
was resorted to, although Mr. Key feared,
at the time, that the mischief proceeded
more from the sloughing of the parts
about the urethra, than from impediment
to the exit of the urine. This opinion
was unfortunately correct, for the infil-
tration extended between the muscles
and peritoneum as high, nearly, as the
diaphragm, shewing that the fascia which
forms the boundary between the peri-
neum and cellular tissue of the pelvis had
sloughed. Under these circumstances,
it was deemed proper to make a free
opening for any matter which might
form, and support the powers of the con-
stitution. This was all that could be
be done, and this was of no avail.
The case is reported in a very credit-
able manner.
Fatal Disease of the Brain
MISTAKEN.
A Gentleman (Mr. B.) aged about 30
years, had a fall from his horse, about
the month of December, 182(5, by which
he was stunned, but from which he soon
revived, and took no farther notice of
the accident. In February of 1827, he
began, for the first time, to feel deep-
seated pains in his head, especially to-
wards the occiput, and, soon afterwards,
sickness at stomach, and even vomiting.
These complaints gradually increased,
and resisted every remedy. In the be-
ginning of September he came up from
the country to London, and went to a
celebrated and eccentric surgeon. He
was then very ill, and his wife told the
surgeon that her husband was dreadfully
afflicted with pain in his head, and sick-
ness at his stomach. " say no more,"
said the surgeon?" I see what is the
matter. Put out your tongue. Take this
prescription, (giving him blue-pill at
night, and black-draught in the morn-
ing,) and go home." He would not hear
a single word of the patient's history, nor
examine into a single symptom except
the state of the tongue. In a week, the
gentleman was much worse, and the wri-
ter of this article was sent for. The coun-
tenance of the patient, the vacant stare
of the eye, and the difficulty of articu-
lating his words, gave strong reason to
suspect disease of the brain. On further
examination, it was found that Mr. Ii.
was totally blind of the left eye, and deaf
of the left ear. There was considerable
difficulty in clothing his ideas in language
?and, indeed, there was also much con-
fusion of thought, and some loss of me-
mory. The pains in the head were ex-
excruciating, and generally produced vo-
miting'once or twice a day. The pulse
was extremely small, and very slow?
sometimes irregular?the body was ema-
ciated?the appetite almost gone?the
bowels torpid. The pupil of the left eye
was more dilated than that of the right,
and little obedient to the light. There
was no paralysis ; but there was great
debility of the limbs, with staggering, on
attempting to walk. There were also
cramps felt in different parts of the
body, which, in a few weeks afterwards,
amounted to convulsions resembling epi-
leptic fits. After this examination, and
receiving the account of the fall, no man
could doubt that the disease was seated
in the head, and that the sickness of sto-
mach was merely symptomatic of the
cerebral disease. The diagnosis and prog-
nosis were stated to his wife, who w;is
greatly shocked at the intelligence, the
surgeon having assured her that it was
merely disorder of the stomach. Counter-
irritation in the neighbourhood of the
head?leechings?cold to the scalp?and
gentle aperient medicines gave a tem-
porary relief, and entirely removed the
gastric irritability, which never after-
wards returned. But in a few weeks the
cramps rose gradually to convulsions?
the emaciation increased?the head-aches
continued?the pulse became as feeble
as a thread?and, on the 5th December,
death put a period to his sufferings. Du-
ring the two months preceding death, he
was daily visited by Mr. Stevenson, sur-
geon, Edgeware Road, and the reporter
only saw him once or twice a week.
On the 6th December the head was
examined. On removing the calvarium
the convolutions were seen to be much
flattened and compressed, which prepared
us for some tumour or hydrocephalic dis-
1828] Sympathetic Apoplexy. 455
tention. The hemispheres were very-
firm, which firmness gradually decreased
as we receded from the surface, and ap-
proached the corpus callosum. This part
?f the brain was very soft. There were
four ounces of clear water in the ven-
tricles. The septum lucidum was gel-
atinous, and there was a large perfora-
tion in the situation of the foramen of
Monro. The iter ad infundibulum was
so enlarged that the finger might he
readily passed down to the sella turcica.
All the parts in this neighbourhood were
ln a state of complete ramollissemcnt
The thalami nervorum were very much
softened, and the tractus opticus was com-
pletely rotten. The, optic nerves, espe-
cially the left one, were greatly wasted.
On taking off the tentorium, a tumour
composed of a cluster of hydatids, ap-
parently with some tubercles intermixed,
vvas seen closely adherent to the pe-
trous portion of the left temporal bone,
and pressing upon the left lobe of the
cerebellum, as well as upon the medulla
oblongata. It had completely annihi-
lated the seventh pair of nerves on that
side?and it had raised up the fifth pair of
nerves, which were seen stretched tightly
over the protuberant morbid mass. It was
about the size of a small pullet's egg.
The loss of hearing is thus readily ac-
counted for on the left side ; but how was
it that we had no distortion, paralysis,
?r loss of feeling in any part of the face,
although the 7th pair of nerves was des-
troyed, and the fifth pair were greatly
stretched and driven out of their natural
situation by the tumour ?
Considering the wanton manner in
^'hich the feelings of patients are sported
with by some medical men?and reflect-
ing on the foregoing sample of indis-
crimination?nay, of positive error, aris-
ing from a preposterous adherence to a
preconceived doctrine that blinds the eye
to every object, we should feel it, in
some degree, criminal to conceal such a
monstrous mode of prescribing for dis-
eases of the most dangerous or fatal na-
ture, without giving one's self the least
trouble to investigate the history, symp-
toms, or seat of the complaint. We hope
this will meet the eye of the surgeon who
so unceremoniously decided on the nature
of the above-mentioned unfortunate gen-
tleman's fatal malady., Hisfeelings would
not be enviable, we imagine, if he suf-
fered himself to reflect, for a moment, on
the terrible consequences which must
often result from such a mode of carrying'
011 the practice of medicine.
Thames Water.
We understand that the Committee of
Inquiry into the purity (God help us!)
of the water supplied from the river to
the inhabitants of this metropolis, is now
sitting. We hope and trust they will do
their duty. We have reason to believe
that active exertions are making to enlist
chemistry against cleanliness ? and to
persuade the town that, " what will not
poison must fattenWe shall keep a
very sharp look out upon the transactions
of this Committee, and we promise to
dissect the results of their inquiry with
the sharpest critical instruments in our
possession.
Sympathetic Apoplexy.
Some people are as tenacious of life as
worms, or as animals which we may di-
vide into pieces without destroying vita-
lity. A gentleman, who had long shewn
symptoms of what Rostan and others
would have termed " Ramollessement thi
Cerveau," fell down, the other day, in a
fit of apoplexy, at the age of 68, and
not the slightest impression was made by
cupping, leeching, blisters, enemas, and
all the means which a trio of physicians
(including Dr. Warren) could suggest.
Mapleson left the patient for dead, after
taking four ounces of blood from the
head ; and he was apparently in articulo
mortis, after 48 hours of general peraly-
sis, total insensibility, stertorous breath-
ing, glassy eyes, and " dead rattles " in
the throat! The physicians parted?to
meet no more?at least in that case. The
ordinary physician took his leave at 12
o'clock at night, requesting to be informed
in the morning, at what hour the patient
died. No message having been sent, the
physician called in the morning, and
found, to his no small surprise, the patient
at his breakfast, quite sensible, and with
the full power of all his muscles ! ! The
patient, soon after this, disgorged some
pints of fetid bile, and had no return of
456 Periscope; or, Circumspective Review. [Jan. 12
apoplectic or paralytic symptoms. This
is one of the many cases, where irritation
of the chylopoietic nerves will simulate
diseases of the most fatal character?and
especially those of the brain and nervous
system generally. Nothing is more com-
mon than partial pyralysis, particularly
in children, from indigestible matters in
the primse vise.; and we are convinced
that many of those apoplectic attacks,
from which we see people quickly re-
cover, without any remaining paralysis,
are dependent on gastric or intestinal
irritation. The foregoing case is a good
example.
LIBERTY OF THE PRESS,
We need hardly appeal to our readers, as
to the fact of our having long and steadily
resisted the calumnies and misrepresen-
tations of the Lancet. For this we have
been the constant theme of vituperation
in that journal, for years past. Every
one knows how Dr. Johnson has been
abused, nick-named, and vilified in the
Lancet; but few are aware that he is
now threatened with the fourth law-suit,
for daring to retaliate on a journal that
is always bellowing forth about the li-
berty of the press, while abusing all,
except the members of its own junto !
The first action was brought against
Dr. J. as Editor of the Mpd.-chir. Re-
view, in the Summer of 1826. A similar
action was brought against the Printer,
for the same libel?and that for the sole
purpose of doubling the law expenses,
for Dr. Johnson never attempted to evade
his responsibility. These two actions
cost Dr. Johnson 7001. A third action
was brought against Mr. Highley, the
publisher, for having sold to Mr. (Fakley
himself, a single copy of the uncancelled
journal, which had been accidentally re-
turned to his shop, as a back number,
from one of the houses in Paternoster
Row ! With a great deal of difficulty,
Mr. Highley got the action compromised,
by paying 32 pounds sterling, which Dr.
Johnson considered himself bound, in ho-
nour, to repay. The fourth action is an-
nounced, for having, in our last Number,
accused the Lancet, as a journal, of fal-
sifying (through its reporters) the cases
that occur in public hospitals?and of
paying the highest price to those repor-
ters that shew most ingenuity in garbling
and distorting those cases occurring un-
der physicians and surgeons, not of the
junto. Mr. Wakley takes all this to him-
self, though we have distinctly absolved
him (and here repeat it) from these
charges, and laid the blame on his re-
porters, his hireling writers?and on some
others of the junto unknown or unseen.
If Mr, Wakley will turn to page 247 of
our last number, he will see, unless his
vanity be much greater than his discern-
ment, that we do not accuse him of brib-
ing, with high fees, the falsifying repor-
ters. " Will it be believed, that there are
men, in this great metropolis, who rank
high in medical science, but who are wicked
enough to expend considerable sums in the
reward of those who can invent the great-
est, but, at the same time, the most plau-
sible falsehoods, against the brightest cha-
racters in the profession." Is it possible
that Mr. Wakley can be vain or shallow
enough to suppose that we mean him in
such passages ? Could he get any man in
London to say, in a court of justice, that
he is the man alluded to ? He knows full
well he could not. In respect to the
charges, then, against the Lancet, as a
publication in which many are concerned,
whose names do not appear, we retract
not one iota of what we have advanced.
If Mr. Wakley will still insist that he him-
self is the Lancet?a surgical instru-
ment, or a medical newspaper?why let
him come into court in a shagreen case
?or neatly folded up and stamped, price
one shilling, and we swear by Apollo?
" Solem quis audeat falsum dicere."
Yea, by every God in Heaven?except
Mercury, the God of false reporters?
and, therefore, perhapsoneof the junto?
that we will put a justification on the
record against the said Lancet, and verify
our accusations by the greatest mass of
evidence that ever came into the King's
Bench or Common Pleas. Yes! and we
will bring into court certain other perso-
nages, who, perchance, may wish them-
selves athousand miles off on that fatal day I
" Give us the opportunity, (to use Mr.
Wakley's own language, towards two of
his ex-editors) kind, modest Editor, of
publicly extracting from these wretches,
(reporters) a history of their own infamy,
andwewill ever after call thee?friend."

				

